# Hormonal Fluctuations during the Estrous Cycle Modulate Heme Oxygenase-1 Expression in the Uterus

**DOI:** 10.3389/fendo.2014.00032

**Published:** 2014-03-13

**Authors:** Maria Laura Zenclussen, Pablo Ariel Casalis, Federico Jensen, Katja Woidacki, Ana Claudia Zenclussen

**Affiliations:** ^1^Department of Experimental Obstetrics and Gynecology, Medical Faculty, Otto-von-Guericke University, Magdeburg, Germany

**Keywords:** heme oxygenase-1, progesterone, estradiol, uterine cells, estrous cycle, mouse

## Abstract

Deletion of the heme oxygenase-1 (HO-1) (*Hmox1*) locus in mice results in intrauterine lethality. The expression of the heme catabolizing enzyme encoded by this gene, namely HO-1, is required to successfully support reproductive events. We have previously observed that HO-1 acts at several key events in reproduction ensuring pregnancy. HO-1 defines ovulation, positively influences implantation and placentation, and ensures fetal growth and survival. Here, we embarked on a study aimed to determine whether hormonal changes during the estrous cycle in the mouse define HO-1 expression that may influence receptivity. We analyzed the serum levels of progesterone and estrogen by ELISA and HO-1 mRNA expression in uterus by real time RT-PCR at the metestrus, proestrus, estrus, and diestrus phases of the estrous cycle. Further, we studied the HO-1 protein expression by western blot upon hormone addition to cultured uterine AN3 cells. We observed that HO-1 variations in uterine tissue correlated to changes in hormonal levels at different phases of the estrus cycle. *In vitro*, HO-1 protein levels in AN3 cells augmented after the addition of physiological concentrations of progesterone and estradiol, which confirmed our *in vivo* observations. Our data suggest an important role for hormones in HO-1 regulation in uterus during receptivity, a process known to have a significant impact in receptivity and later on blastocyst implantation.

## Introduction

Heme oxygenases (HOs) catalyze heme catabolism toward biliverdin, carbon monoxide (CO), and free iron (1). The stress-responsive heme oxygenase-1 (HO-1) isoform, encoded by the *Hmox1* gene, is cytoprotective and modulates anti-inflammation [(2); reviewed in Ref. (3, 4)] as well as cell proliferation preventing tissue injury and regulating innate as well as adaptive immune responses (5).

During normal pregnancy, complex processes take place in a defined succession, each of them being unique. Pregnancy begins with the fertilization of the ovum, followed by implantation of the blastocyst in the maternal uterus. To implant, the blastocyst needs to adhere to the endometrium and be provided with oxygen and nutrients. For these dramatic changes to occur, uterine tissue remodeling and inflammatory processes are required. As the ablation of immunosuppressive molecules is detrimental, it has to be assumed that both inflammatory and anti-inflammatory pathways are required (6, 7). HO-1 seems to be a crucial player interfering with many – if not all – of these sequenced processes. We have recently showed that HO-1 defines ovulation (8) and is critical for pregnancy success, regulating proper implantation, placentation, and intrauterine fetal survival (7). After implantation occurred and while placentation is taking place, a period of immune tolerance must exist that allows the half-foreign fetus to grow without being attacked by the maternal immune system. Also at this step, HO-1 is of importance. It modulates the maternal immune system to allow tolerance toward the growing fetus by affecting the function of dendritic cells and regulatory T cells (9, 10). Hence, HO-1 is a central regulator of pregnancy as it critically interferes with important steps namely ovulation, implantation, placentation, fetal development, and immune tolerance. Poor reproductive outcome of *Hmox1*-deficient animals may however be additionally due to defects in receptivity upon hormonal changes.

As nidation and later implantation much depends on the uterine receptivity (11) and we observed that both processes are defective in the absence of HO-1 (7), we hypothesized that normal hormonal changes during the estrous cycle would modulate HO-1 expression. At receptivity, the uterine tissue undergoes dramatic changes, all of them preparing the uterus for a possible nidation of the fertilized egg. HO-1 might be implied in tissue protection during receptivity because of its known roles as anti-inflammatory, tissue-protective, and anti-oxidant enzyme (12). Here, we show that hormonal changes as observed at the different phases of the estrous cycle provoke important variations on the levels of HO-1 *in vivo*. HO-1 peaks at receptivity and significantly correlates with the progesterone peak. *In vitro*, combined estrogen and progesterone application leads to increased HO-1 protein expression in uterine cells. Thus, hormonal changes during the estrous cycle dictate HO-1 uterine levels. Our data contributes to the understanding of how hormones condition the uterine environment to be prepared for nidation of the fertilized egg.

## Materials and Methods

### Determination of the phases of the estrous cycle

For testing the different phases of the estrous cycle in *Hmox1*^+/+^ animals, vaginal smears were obtained daily and stained as explained elsewhere (13). HO-1 mRNA levels were measured in uterus and spleen normalized to beta-actin by using TaqMan technology as described below.

### Hormone measurements

Levels of progesterone and estradiol were analyzed in plasma samples from wild type females at different stages of their estrous cycle (BALB/c *Hmox1*^+/+^ mice). Progesterone was measured by LIA (chemiluminescence immunoassay) using a kit from Diagnostics Biochem Canada Inc., ON, Canada. RLUs were measured in microplate luminometer (Micromat Plus LB 96V) at 0.1 and 2 s and the values were expressed as RLU/s. Estradiol levels were measured by ELISA using a kit from Diagnostics Biochem Canada Inc., using a microplate well reader at 450 nm following the manufacturer’s instructions.

### Heme measurement

Heme was measured in uterine lavage. Briefly, uteri of the females were flushed with 150 ml of saline solution. Plasma and lavage were both passed through a Microcon YM-3 column (Millipore; 90 min at 14°C, 16,000 × *g*) to remove proteins (MW > 3 kDa). We quantified free heme in protein-depleted uterine lavage using a chromogenic assay according to the manufacturer’s instructions (QuantiChrom heme assay kit, Bioassay Systems).

### RNA isolation and cDNA synthesis

RNA extraction was performed using Trizol reagent. Briefly, tissues (spleen, uterus, liver) were homogenized in Trizol using an Ultra-Turrax T25 homogenizer. After adding chloroform and vortexing for 2 min at RT, samples were centrifuged at 10,000 × *g* for 10 min at 4°C. The upper phase obtained after the centrifugation was then transferred to a new tube, and ice-cold ethanol was added. After an incubation of 10 min at −20°C, samples were centrifuged for 10 min at 10,000 × *g* at 4°C. The pellet obtained after this centrifugation was then washed three times with ethanol 80° and between each wash step, cells were centrifuged for 10 min at 10,000 × *g* at 4°C. After the last wash, the pellet was allowed to dry and it was re-suspended with RNase-free water. RNA concentration was determined by measuring OD at 260 nm. For cDNA synthesis, samples containing 2 μg of total RNA were placed for 2 min on ice and added with dNTPs [(2.5 mM), Amersham Pharmacia, Munich, Germany], DNase I (2 U/μl, Stratagene, Waldbronn, Germany), and RNase-inhibitor (40 U/μl) mixed in reaction buffer. The mix was incubated for 30 min at 37°C and further heated to 75°C for 5 min. The addition of the reverse transcriptase (200 U/μl) and RNase-inhibitor in distilled water started the reverse transcription. This reaction mixture was incubated at 42°C for 60 min followed by incubation at 94°C for 5 min. Once the cDNA synthesis was completed, the samples were immediately used or kept at −20°C.

### Real time PCR

For HO-1 amplification, TaqMan technology was employed as described elsewhere (14). One microliter of cDNA was used as starting volume to amplify the DNA. PCR-Mastermix (6.25 μl; Eurogentec, Cologne, Germany), 3 μl of the primer mix, 0.5 μl of the fluorescent probes, and RNase-free water were added to a final volume of 13 μl. The amplification reactions were performed on the ABI Prism 7700 sequence detection system (PerkinElmer Applied Biosystems, Darmstadt, Germany) as follows: 2 min at 50°C, followed by an initial denaturation step of 10 min at 95°C, and 40 cycles of 15 s at 95°C and 1 min at 60°C. β-actin was used as housekeeping gene.

### Culture of uterine cells and treatment with hormones

The human uterine cell line (AN3), which is representative of the non-receptive phase of the uterine tissue (15, 16), was purchased from the American Type Culture Collection (ATCC, Wesel, Germany). Cells were maintained in MEM medium (Life Technology, Darmstadt, Germany) supplemented with FBS (10%, Biochrom, Berlin, Germany), 1% of non-essential amino acids (NEAA), 1 mM sodium pyruvate, and antibiotics (Life Technology, Darmstadt, Germany). For hormonal treatment experiments, 5 × 10^5^ cells were cultured for 24 h on a 24 well-plate with 1 ml of MEM medium without phenol red and supplemented with 3% of charcoal-stripped fetal bovine serum and antibiotics. Afterward, cells were treated with water-soluble estradiol (100 ng/ml) and progesterone (10 pg/ml) (both from Sigma-Aldrich, Taufkirchen, Germany) for 24 h. These concentrations were chosen as they represent the physiological values found in healthy women at ovulation.

### Protein isolation

Protein isolation and quantification were done following a standard protocol established in our laboratory (17). AN3 cells were re-suspended and homogenized in lysis buffer (1% NP-40, 0.1 mg/ml *n*-dodecyl beta maltoside, 10 mM NAO3V, 1 M Tris pH 7.5, 5 m NaCl, 500 mM NaF, 500 mM EDTA pH 7.5, 100 mM PMSF) for 45 min. Homogenates were centrifuged at 12,000 rpm for 20 min at 4°C and the supernatant containing the proteins was collected. Protein was isolated from tissue samples (uterus) from the phenol phase from the Trizol RNA isolation. Protein concentration was assessed by using the Pierce BCA protein assay (Thermo Fisher Scientific, Bonn, Germany) as indicated by the manufacturer. Protein samples were kept at −80°C until use.

### Western blot

For western blot analysis, 20 μg of proteins was transferred into a 12% polyacrylamide gel and a SDS-PAGE in denaturizing conditions was performed at 100 V. After the electrophoresis, proteins were transferred into PVDF membranes in transfer buffer containing 20% methanol (v/v), 0.19 M glycine, and 0.025 M Tris-base of pH 8.3. For protein detection, membranes were incubated overnight (ON) with a specific rabbit anti-human HO-1 antibody (Enzo Life Science, Lörrach, Germany). After three washing steps with TBST (TBS with 0.5% Tween) for 5 min each, the membranes were incubated with an anti-rabbit HRP-conjugated (Thermo Fisher Scientific, Schwerte, Germany) antibody diluted 1:2000 for 1 h at RT and then with avidin–horseradish peroxidase complex (ABC complex, Biozol, Eching, Germany). GAPDH was used as loading control in the same gel and applied together with the HO-1 antibody. The chemiluminescence signal was generated by using luminol (A8511-5G, Sigma-Aldrich), 4-hydroxycinnamic acid (*p*-coumaric acid; C9008-25G, Sigma-Aldrich), and hydrogen peroxide (Merck, Darmstadt, Germany). The intensity of the bands was quantified by using the GeneSnap^®^ Software, Version 4.01c from Syngene, Darmstadt, Germany.

### Statistics

All data presented in this manuscript are normally distributed and shown as mean ± SD. The number of animals included in each experiment is specified in the figures. Differences among two groups were analyzed by paired *t*-test using two-way ANOVA and applied to compare three or more groups. All analyses were performed using Graph Pad (^#^*p* < 0.1 and *p < 0.05). The non-parametric Spearman correlation was used to analyze the correlation of RT-PCR and ELISA data.

## Results

### Serum progesterone levels peak during estrus of the estrous cycle

**Figure 1:**
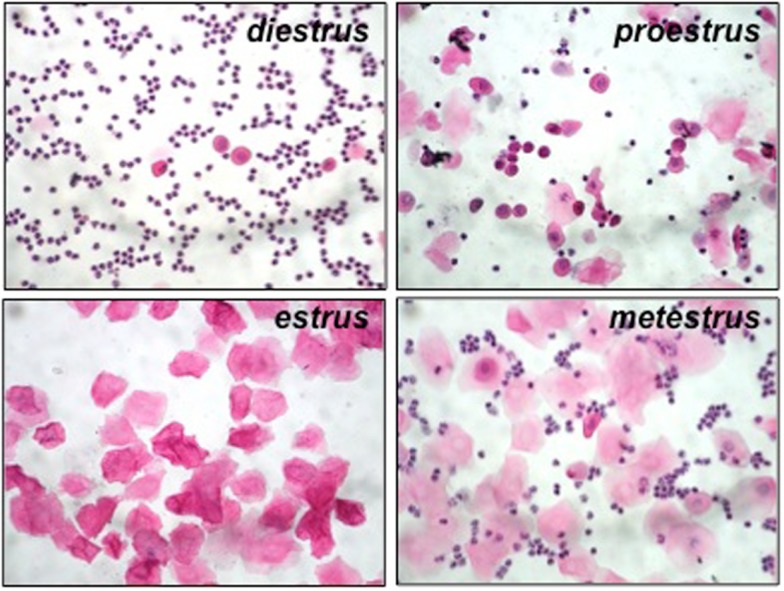
**Representative pictures from hematoxylin/eosin-stained samples from vaginal lavage at each phase of the estrous cycle in *Hmox1*^+/+^ BALB/c female mice**. At diestrus, only leukocytes can be observed, accompanied by few epithelial nucleated cells. At proestrus, mostly nucleated and few cornified cells are present along with some leukocytes. In estrus (at receptivity), only cornified cells are present. At metestrus, cornified epithelial cells and leukocytes are present.

We first determined the phase of the estrous cycle in virgin mice by analyzing the cellular content of their vaginal lavage after staining with hematoxylin/eosin. At proestrus, mostly nucleated and few cornified cells are present along with some leukocytes. In estrus, only cornified cells are present. At metestrus, cornified epithelial cells and leukocytes are present while at diestrus, which is the longest phase, only leukocytes are seen accompanied by few epithelial nucleated cells (Figure 1).

**Figure 2:**
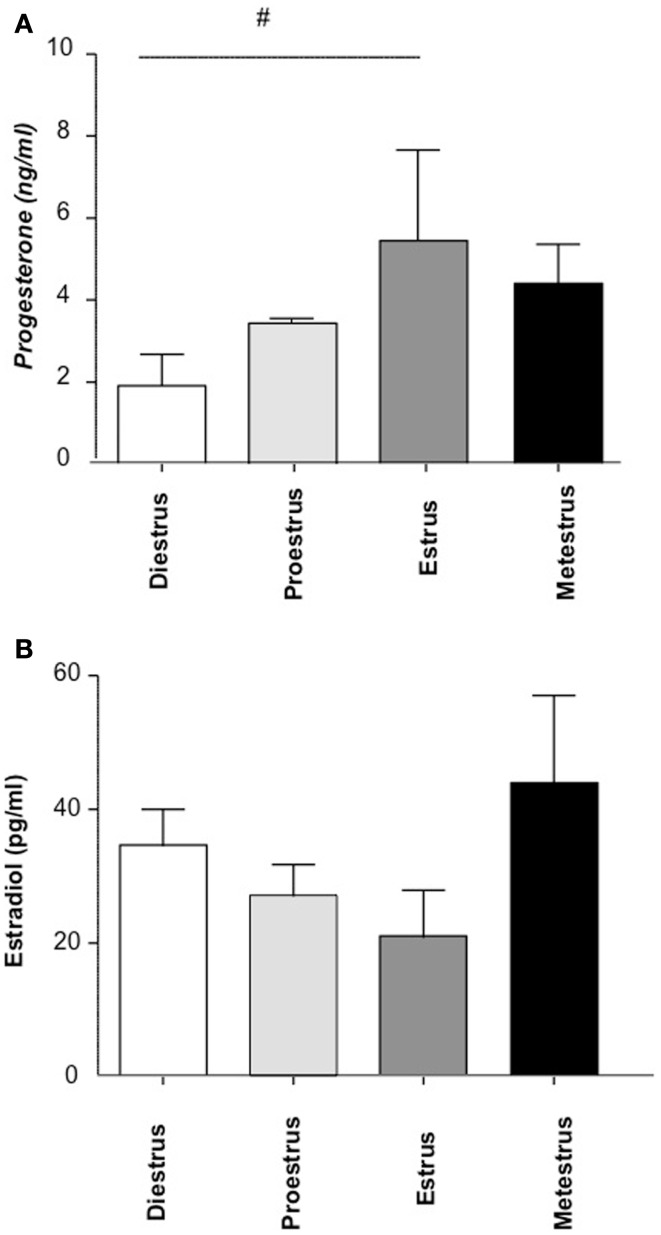
**The concentration of progesterone (A) and estradiol (B) during the estrous cycle (*n* = 16, 4/cycle)**. Progesterone and estradiol levels in plasma were analyzed by ELISA following the instructions of the manufacturer. *n* = 4 mice/phase were included and the statistic analysis was performed by two-way ANOVA.^#^*p* < 0.1.

To quantify hormone levels (progesterone and estradiol), we employed ELISA assays with *n* = 5 animals/phase. We observed, as expected, a peak in progesterone levels during estrus, associated with a decrease in estradiol (Figures 2A,B, respectively).

### HO-1 uterine expression peaks during receptive period

**Figure 3:**
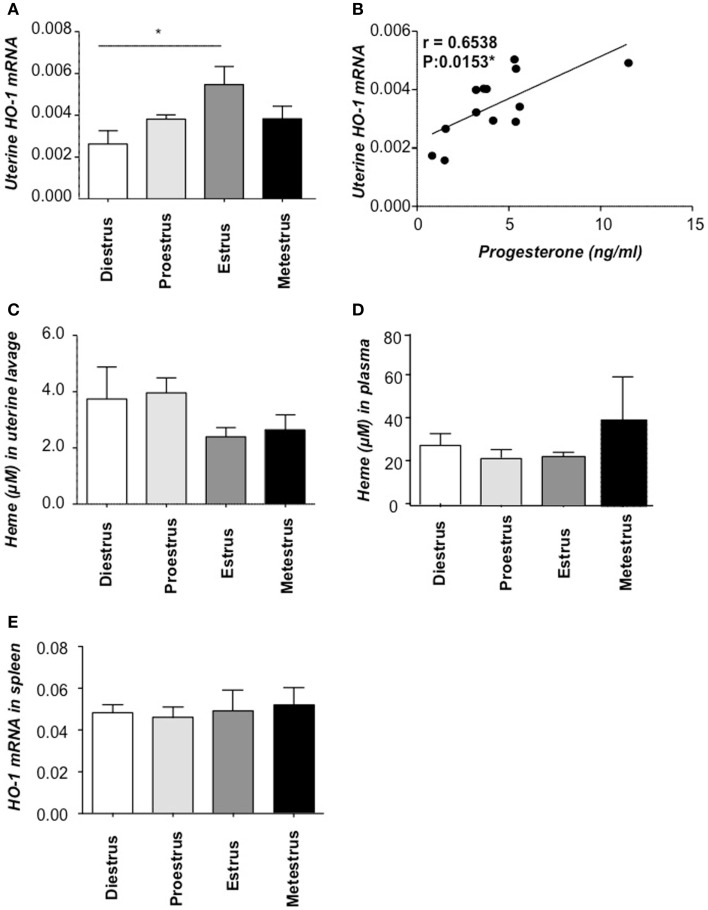
**HO-1 and heme levels at different phases of the estrous cycle**. **(A)** HO-1 mRNA levels in uterine tissue of *Hmox1*^+/+^ female mice (*n* = 4/phase) and **(B)** the positive correlation between HO-1 mRNA levels in the uterus and progesterone concentrations in serum. **(C)** The levels of heme in uterine lavage and **(D)** heme levels in plasma. **(E)** HO-1 mRNA levels in spleen of *Hmox1*^+/+^ female mice. *p < 0.05, *n* = 4 mice/phase were included and the statistic analysis was performed by two-way ANOVA.

We next asked whether HO-1 expression in the uterus of wild type (*Hmox1*^+/+^) mice of BALB/c background would be regulated by hormonal changes during the estrous cycle. That would indicate that HO-1 is implied in female sexual receptivity that occurs during the estrus period of the estrous cycle in which ovulation takes place (18). There was a slight but significant increase in the level of *Hmox1* mRNA expression in the transition from diestrus to proestrus phase of the estrous cycle (Figure 3A), followed by stronger increase thereafter, reaching maximal levels at the estrus phase (Figure 3A), thus coinciding with the phase of the estrous cycle associated with maximal receptivity (18). Interestingly, the mRNA HO-1 peak was associated with the progesterone peak and there was a statistically significant positive correlation among these two (Figure 3B). At protein level, HO-1 was augmented in estrus compared to proestrus and metestrus (Figure S1 in Supplementary Material). Accordingly, the levels of free heme, the HO-1 substrate, in uterine lavage were diminished at this time point, albeit not statistically significant (Figure 3C) while they were unaltered in spleen (Figure 3D). Levels of *Hmox1* mRNA expression normalized to beta-actin in spleen showed no significant changes during the cycle (Figure 3E). Our data suggest that the interaction HO-1/hormones is unidirectional, as hormones clearly influence HO-1 expression as shown here but HO-1 or its absence does not seem to influence hormone levels (7).

### Application of physiological concentrations of progesterone plus estradiol provoked an up-regulation of HO-1 protein levels in AN3 cells

**Figure 4:**
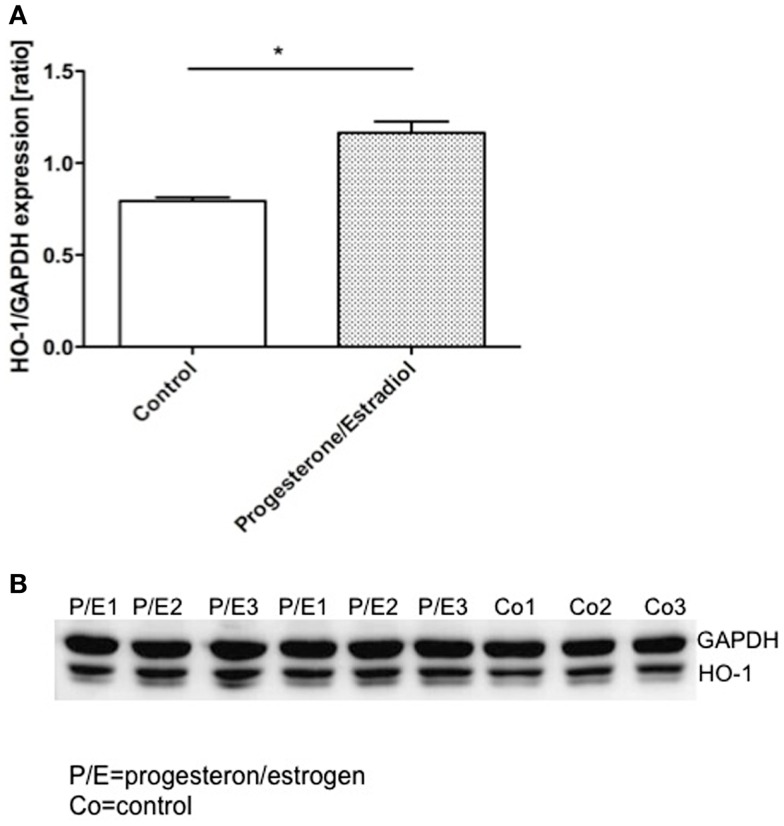
**The representative results of three *in vitro* experiments in which AN3 cells were cultured in the presence or absence of estrogen (E_2_) and progesterone (P_4_) for 24 h**. **(A)** The levels of HO-1 relative to GAPDH were elevated in the cells treated with hormones compared to the controls. **(B)** Representative western blots. The data are expressed as mean ± SEM. *p < 0.05; *n* = 4, the statistic analysis was performed by two-way ANOVA.

To certainly confirm the modulation of uterine HO-1 by hormones, we next studied their effect on HO-1 protein expression in uterine cells, namely AN3 (15). We observed that the addition of combined progesterone and estradiol provoked a significant augmentation in the HO-1 expression after 24 h (Figure 4). This was not a result of cellular stress due to the culture as HO-1 did not augment in cells cultured without hormones (Figure 4). Thus, HO-1 expression in uterine cells depends on female hormones.

## Discussion

Heme oxygenase is a ubiquitous enzyme that catalyzes the initial and rate-limiting steps in the oxidative degradation of heme to bilirubin (1). HO cleaves a mesocarbon of the heme molecule, producing equimolar quantities of biliverdin, iron, and carbon monoxide (CO). CO and biliverdin, both generated via the catabolism of heme by the isoform HO-1, are potent immunosuppressors [revised in Ref. (2, 12)]. HO-1 emerges as a critical regulator of pregnancy success as it determines intrauterine fetal survival. HO-1 positively influences placentation and fetal growth by avoiding the toxic effects of free heme (7). It seems that the presence of HO-1 avoids the accumulation of free heme that is toxic for the tissues (7). As at the fetal–maternal interface the amount of circulating blood is enormous, it is clear that the inadequate clearance of hemoglobin-derived free heme has pathologic consequences. Not only is HO-1 protective because of main activity, which is to catabolize free heme, but also it has been shown that HO-1 positively influences the adaptive immune system and provokes a bias toward a protective response that was highlighted in several inflammation models (12). In fact, we found that later in pregnancy, when immunological tolerance is needed, HO-1 modulates the maternal immune system to allow tolerance toward the growing fetus (9, 10). Hence, HO-1 acts at different levels and different time points of pregnancy to support implantation, placentation, fetal growth, and immune tolerance.

It is known that hormones in general and progesterone in particular prepare the uterus for nidation (19). Estrogen and progesterone are necessary for implantation of the blastocyst, and the preimplantation estrogen surge is essential for implantation in mice. As HO-1 is a molecule that avoids tissue damage because of excessive inflammation and implantation is an inflammatory process, it is possible that HO-1 is implied at this stage. As the subfertility of *Hmox1*^±^ and *Hmox1*^−/−^ mice can have multiple causes despite the ones we already found (7, 8), we asked here whether the HO-1 expression in the uterus of wild type (*Hmox1*^+/+^) mice may be regulated by hormones controlling female sexual receptivity. HO-1 mRNA expression reached its maximal level at the estrus phase, thus coinciding with maximal receptivity (18). Accordingly, the levels of heme, the HO-1 substrate, in uterine lavage were reduced at this time point, confirming increased HO-1 activity. Interestingly, HO-1 peak in the uterus coincided with the augmentation of progesterone in serum during estrus phase, this positive correlation being statistically significant. Thus, it is tempting to speculate that the expression of uterine HO-1 is regulated by hormonal changes during the estrous cycle. HO-1 expression might influence sexual receptivity in the uterus thus regulating the frequency of pregnancies when mating, e.g., *Hmox1*^±^ mice to maintain the colony. Uterine receptivity implies the highest progesterone and the highest HO-1 in the uterine tissue; this would protect it from excessive inflammation at nidation of the egg and posterior implantation. Here, we found that expression of HO-1 peaks during the receptive period. This was inverse to what we observed for the heme concentration, which indicates that heme/HO-1 dictate the receptivity in the uterus and would in part explain the lower frequencies of pregnancies in a period of 1 year in mice partially deficient in HO-1 (unpublished observations). The direct effect of hormones, progesterone and estrogen, on the expression of uterine HO-1 was confirmed *in vitro* in uterine AN3 cells. This strengthens the hypothesis of hormones dictating the concentration of HO-1, a cytoprotective molecule, in uterine tissue.

Cella and colleagues described that the application of estrogens and progesterones to ovariectomized rats provoked an increase in HO-1 expression (20). The authors interpreted these results as HO-1 being important for defense against oxidative stress. Progesterone modulation of uterine HO-1 by hormones may also be relevant to maintain uterine quiescence as HO-1 induction limited uterine contractility in pregnant myometrium (21).

From our published work, we know that HO-1 expression in oocytes promotes ovulation, probably by affording cytoprotection during the process of follicular rupture, a proinflammatory event associated with tissue injury, including hemolysis (8). HO-1 further influences positively trophoblast survival and differentiation from stem cells into giant cells, it influences the attachment of the blastocyst to the uterine epithelial wall and thus the quality of implantation. As a consequence, we found HO-1 to be important for placentation and fetal growth (7). HO-1 later modulates cells of the innate and adaptive immune system to have a pregnancy-supporting tolerant immune response. Here, we show that additionally to all these pregnancy-fostering actions, hormones modulate HO-1 expression so it can be highly expressed at receptivity in the uterus. Our data add one more piece to the fascinating puzzle that is pregnancy establishment and support.

## Author Contributions

Maria Laura Zenclussen, Pablo Ariel Casalis, Federico Jensen, and Katja Woidacki performed experiments. Ana Claudia Zenclussen, Pablo Ariel Casalis, Federico Jensen, and Katja Woidacki designed and analyzed experiments. Maria Laura Zenclussen and Ana Claudia Zenclussen wrote the paper. Ana Claudia Zenclussen supervised the work and provided the funding to perform the study.

## Conflict of Interest Statement

The authors declare that the research was conducted in the absence of any commercial or financial relationships that could be construed as a potential conflict of interest.

## Supplementary Material

The Supplementary Material for this article can be found online at http://www.frontiersin.org/Journal/10.3389/fendo.2014.00032/abstract

Figure S1 **Western Blots for HO-1 and GAPDH performed with uterus samples from animals at their proestrus, estrus and metestrus phase of the estrous cycle (*n* = 4/each)**. At the bottom, the ratio of HO-1/GAPDH is expressed as mean ± SEM.

## References

[1] Tenhunen R Marver HS Schmid R The enzymatic conversion of heme to bilirubin by microsomal heme oxygenase. Proc Natl Acad Sci U S A (1968) 61:748–55 10.1073/pnas.61.2.748 4386763 PMC225223

[2] Otterbein LE Bach FH Alam J Soares M Tao Lu H Wysk M Carbon monoxide has anti-inflammatory effects involving the mitogen-activated protein kinase pathway. Nat Med (2000) 6:422–8 10.1038/74680 10742149

[3] Otterbein LE Soares MP Yamashita K Bach FH Heme oxygenase-1: unleashing the protective properties of heme. Trends Immunol (2003) 24:449–55 10.1016/S1471-4906(03)00181-9 12909459

[4] Soares MP Marguti I Cunha A Larsen R Immunoregulatory effects of HO-1: how does it work? Curr Opin Pharmacol (2009) 9:482–9 10.1016/j.coph.2009.05.008 19586801

[5] Soares MP Bach FH Heme oxygenase-1: from biology to therapeutic potential. Trends Mol Med (2009) 15:50–8 10.1016/j.molmed.2008.12.004 19162549

[6] McLennan IS Koishi K Fetal and maternal transforming growth factor-beta 1 may combine to maintain pregnancy in mice. Biol Reprod (2004) 70:1614–8 10.1095/biolreprod.103.026179 14766723

[7] Zenclussen ML Casalis PA El-Mousleh T Rebelo S Langwisch S Linzke N Haem oxygenase-1 dictates intrauterine fetal survival in mice via carbon monoxide. J Pathol (2011) 225:293–304 10.1002/path.2946 21744344

[8] Zenclussen ML Jensen F Rebelo S El-Mousleh T Casalis PA Zenclussen AC Heme oxygenase-1 expression in the ovary dictates a proper oocyte ovulation, fertilization, and corpora lutea maintenance. Am J Reprod Immunol (2012) 67:376–82 10.1111/j.1600-0897.2011.01096.x 22133191

[9] Zenclussen ML Anegon I Bertoja AZ Chauveau C Vogt K Gerlof K Over-expression of heme oxygenase-1 by adenoviral gene transfer improves pregnancy outcome in a murine model of abortion. J Reprod Immunol (2006) 69:35–52 10.1016/j.jri.2005.10.001 16386310

[10] Schumacher A Wafula PO Teles A El-Mousleh T Linzke N Zenclussen ML Blockage of heme oxygenase-1 abrogates the protective effect of regulatory T cells on murine pregnancy and promotes the maturation of dendritic cells. PLoS One (2012) 7:e42301 10.1371/journal.pone.0042301 22900010 PMC3416808

[11] Sandra O Mansouri-Attia N Lea RG Novel aspects of endometrial function: a biological sensor of embryo quality and driver of pregnancy success. Reprod Fertil Dev (2011) 24:68–79 10.1071/RD11908 22394719

[12] Gozzelino R Jeney V Soares MP Mechanisms of cell protection by heme oxygenase-1. Annu Rev Pharmacol Toxicol (2010) 50:323–54 10.1146/annurev.pharmtox.010909.105600 20055707

[13] Parkes AS The role of the corpus luteum in the maintenance of pregnancy. J Physiol (1928) 65:341–9 16993955 PMC1515064

[14] Zenclussen AC Gerlof K Zenclussen ML Sollwedel A Bertoja AZ Ritter T Abnormal T-cell reactivity against paternal antigens in spontaneous abortion: adoptive transfer of pregnancy-induced CD4+CD25+ T regulatory cells prevents fetal rejection in a murine abortion model. Am J Pathol (2005) 166:811–22 15743793 10.1016/S0002-9440(10)62302-4 PMC1602357

[15] Dawe CJ Banfield WG Morgan WD Slatick MS Cuth HO Growth in continuous culture, and in hamster, of cells from a neoplasm associated with acanthosis nigricans. J Natl Cancer Inst (1964) 33:441–56 14207855

[16] Ho H Singh H Aljofan M Nie G A high-throughput in vitro model of human embryo attachment. Fertil Steril (2012) 97:974–8 10.1016/j.fertnstert.2012.01.116 22341638

[17] El-Mousleh T Casalis PA Wollenberg I Zenclussen ML Volk HD Langwisch S Exploring the potential of low doses carbon monoxide as therapy in pregnancy complications. Med Gas Res (2012) 2(1):4 10.1186/2045-9912-2-4 22348450 PMC3837472

[18] Edwards DA Pfeifle JK Hormonal control of receptivity, proceptivity and sexual motivation. Physiol Behav (1983) 30:437–43 10.1016/0031-9384(83)90150-6 6683412

[19] Chaen T Konno T Egashira M Bai R Nomura N Nomura S Estrogen-dependent uterine secretion of osteopontin activates blastocyst adhesion competence. PLoS One (2012) 7:e48933 10.1371/journal.pone.0048933 23152823 PMC3494704

[20] Cella M Farina MG Keller Sarmiento MI Chianelli M Rosenstein RE Franchi AM Heme oxygenase-carbon monoxide (HO-CO) system in rat uterus: effect of sexual steroids and prostaglandins. J Steroid Biochem Mol Biol (2006) 99:59–66 10.1016/j.jsbmb.2005.11.007 16524721

[21] Acevedo CH Ahmed A Hemeoxygenase-1 inhibits human myometrial contractility via carbon monoxide and is upregulated by progesterone during pregnancy. J Clin Invest (1998) 101:949–55 10.1172/JCI927 9486963 PMC508644

