# Supplementary figures and images for: Hormonal Fluctuations during the Estrous Cycle Modulate Heme Oxygenase-1 Expression in the Uterus
Source: Front Endocrinol (Lausanne). 2014 Mar 13;5:32. doi: 10.3389/fendo.2014.00032 (PMC3952397; doi:10.3389/fendo.2014.00032)

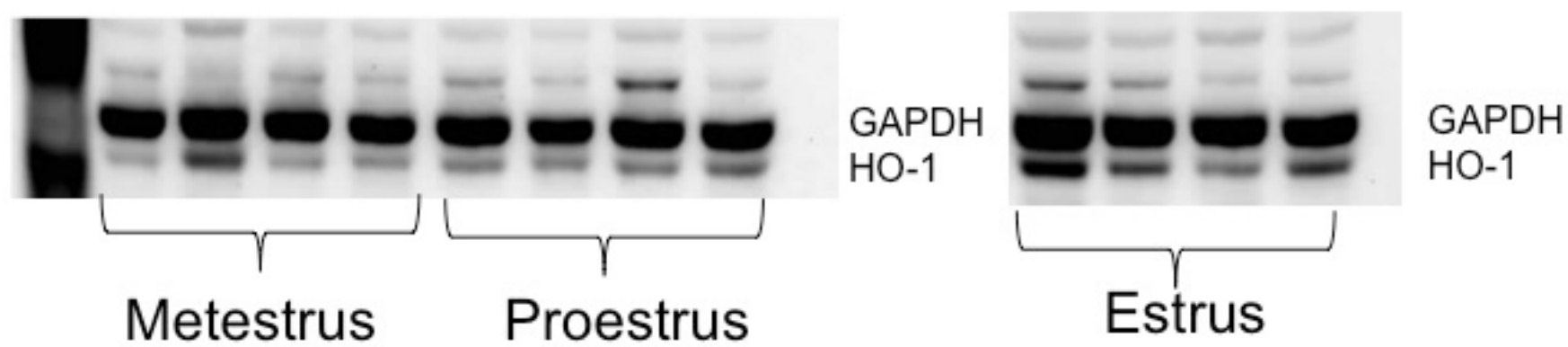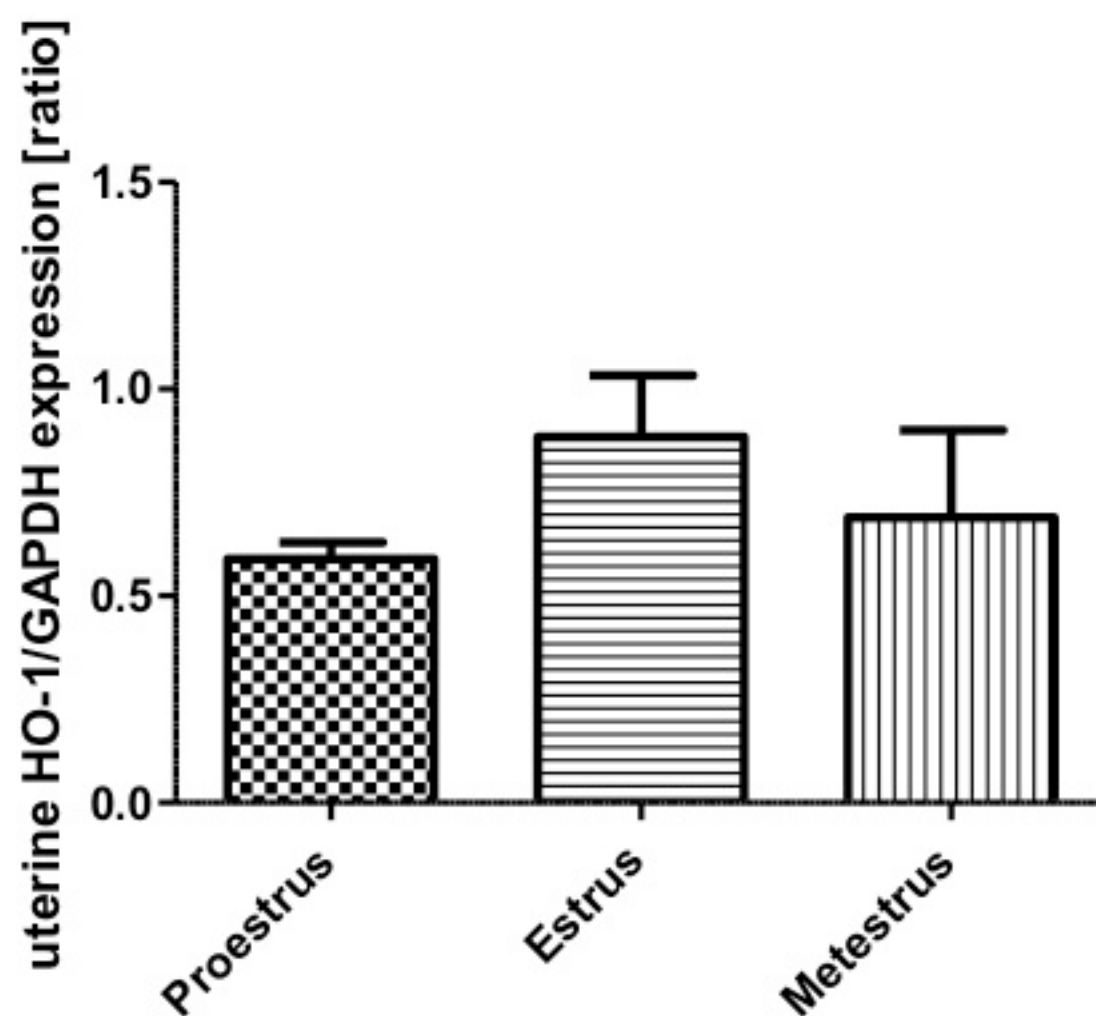

***Supplementary Figure 1***

Supplement: Figure S1 — Western Blots for HO-1 and GAPDH performed with uterus samples from animals at their proestrus, estrus and metestrus phase of the estrous cycle (n = 4/each). At the bottom, the ratio of HO-1/GAPDH is expressed as mean ± SEM. [file Presentation1.PDF]
